# Plant-Mediated Fabrication and Surface Enhanced Raman Property of Flower-Like Au@Pd Nanoparticles

**DOI:** 10.3390/ma7021360

**Published:** 2014-02-19

**Authors:** Daohua Sun, Genlei Zhang, Jiale Huang, Haitao Wang, Qingbiao Li

**Affiliations:** Department of Chemical and Biochemical Engineering, College of Chemistry and Chemical Engineering, Xiamen University, Xiamen 361005, Fujian, China; Emails: genleizhang@tju.edu.cn (G.Z.); cola@xmu.edu.cn (J.H.); wht@xmu.edu.cn (H.W.); kelqb@xmu.edu.cn (Q.L.)

**Keywords:** bioreduction, core-shell, *Cacumen Platycladi*, nanoflowers

## Abstract

The flower-like nanostructures of an Au core and Pd petals with the average size of 47.8 nm were fabricated through the successive reduction of HAuCl_4_ and Na_2_PdCl_4_ at room temperature. During the synthesis, *Cacumen Platycladi* leaf extract served as weak reductant and capping agent. Characterization techniques such as Energy-dispersive X-ray spectroscopy, UV-Vis spectroscopy, and X-ray diffraction characterizations were employed to confirm that the as-synthesized nanoparticles have the structure of core-shell. The obtained core-shell nanoflowers exhibited good surface enhanced Raman spectroscopic activity with Rhodamine 6G.

## 1. Introduction

Multi-metallic nanoparticles (NPs) with alloy or core-shell structures are attractive materials owing to their composition-dependent optical, catalytic, electronic, and magnetic properties which are distinctly different from those of their monometallic counterparts [1–3]. As in the case of monometallic NPs, these properties can be controlled by tuning the shape and size of the NPs. Accordingly, shape-controlled synthesis of bimetallic NPs, especially the core-shell structure, has been extensively studied in efforts to optimize their properties [4,5]. To date, except for a few reports on one-step synthesis [6], core-shell bimetallic NPs are generally prepared by seeding-growth method where pre-synthesized metal NPs are used as seeds for the overgrowth of the second metal [7]. According to this method, various core-shell bimetallic NPs, such as Ru@Pt [8], Au@Ag [9] and Au@Pd [10] have been successfully synthesized by choosing different metal NPs as seeds. Notably, the seeding-growth method has been widely used to prepare bimetallic core-shell NPs with well-defined geometries, wherein a second metal layer is grown over the pre-synthesized seed NPs [11,12]. However, most of studies on the synthesis of alloy or core-shell nanostructures have concentrated on isotropic spherical structures. Very few methods have been reported for fabricating alloy or core-shell structures with anisotropy, non-spherical shape [13]. Indeed, the synthesis of NPs with controlled shapes has been a subject of intense research in recent years because their specific geometries lead to unusual physical and chemical properties [14,15] and they can be promising building blocks for the creation of nanostructured materials [16].

The seeding-growth method is a relatively easy method to obtain core-shell structures, especially in nanomaterials because of their high surface energy and deviation from the equilibrium status [9]. However, the method utilizes not only chemical reductants but also auxiliary stabilizers, and often requires specialized and expensive equipments. With the problem of the energy crisis and its constraint being particularly concerning to developing economies, the need to seriously consider alternative traditional chemistry has received a significant boost through the efforts of multidisciplinary and interdisciplinary scientific fields. Hence, the development of efficient green chemistry methods for synthesis of metal NPs has become a major focus of researchers. Biological resources available in nature, such as bacteria, fungi, yeast and plants have been reported for the synthesis of a variety of metal NPs conducted at room temperature [17]. Since the maintenance of cell cultures is elaborate, plants seem to be the best candidate. They are believed to act as reducing agents and stabilizing agents during the synthesis process [18]. Accordingly, there is a renewed trend in employing plants to synthesize bimetallic NPs [19–22]. However, regrettably, so far, to the best of our knowledge, almost all of this work was based solely on alloy NPs, such as Au-Ag and Au-Pd, while NPs with core-shell structure through biosynthesis were barely investigated. Herein we report a facile and green route for synthesizing flower shaped Au-Pd core-shell NPs at room temperature. Characterization techniques such as transmission electron microscopy (TEM), ultraviolet-visible spectroscopy (UV-Vis), energy dispersive X-ray spectroscopy (EDX), selected area electron diffraction (SAED) and X-ray diffraction (XRD) were employed. Moreover, the flower-like Au-Pd alloy NPs exhibited desired surface enhanced Raman spectroscopic (SERS) activity with Rhodamine 6G (R6G).

## 2. Results and Discussions

### 2.1. Synthesis of Flower-Like Au-Pd Core-Shell Nanoparticles

**Figure 1.**
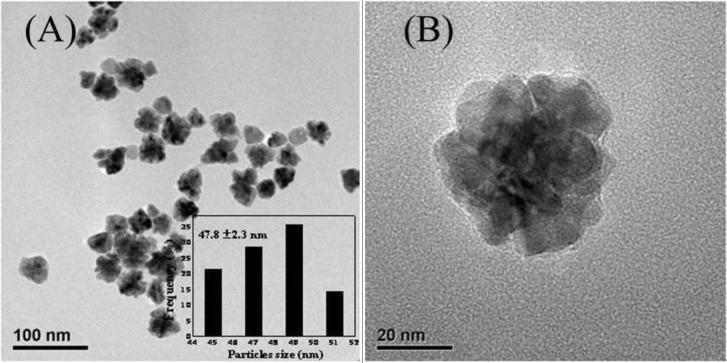

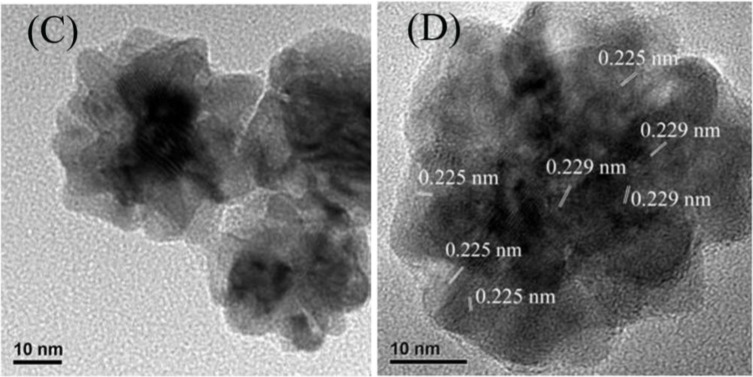
(**A**) TEM image, (**B**–**D**) High-resolution TEM (HRTEM) images displaying the lattice fringes of as-prepared NPs with initial Au/Pd molar ratio of 1:1. The inset in (**A**) indicates the size distribution of the NPs.

**Scheme 1.**
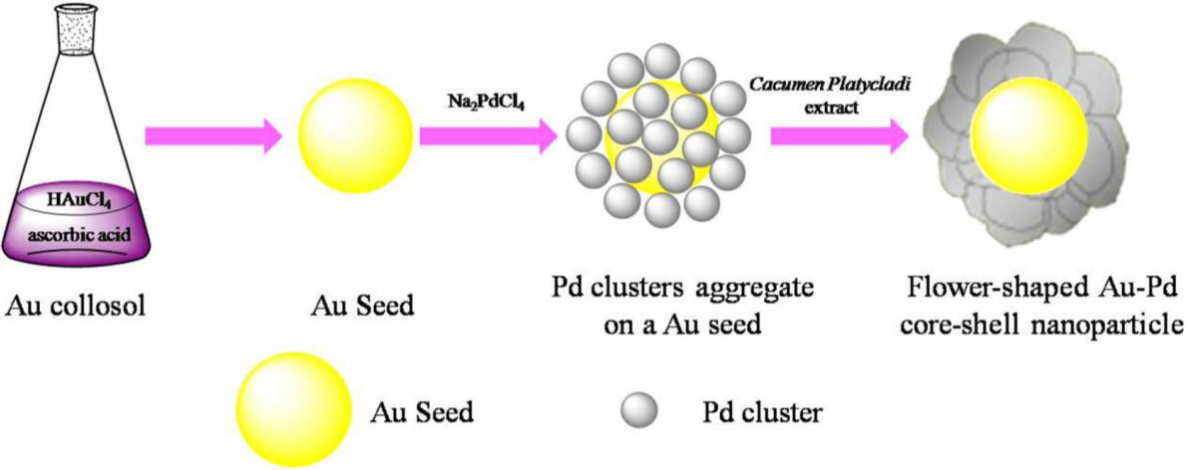
Model proposed for the formation of flower shaped Au-Pd core-shell NPs.

The success of deposition of one metal on the preformed NPs of another metal to form a core-shell structure depends on the reducing reagent and preparation conditions strongly. Scheme 1 depicted the formation of flower shaped Au@Pd nanoparticles. Firstly, Au seeds were formed by the fast reduction of chloroauric acid (HAuCl_4_) by Ascorbic acid (AA). Afterwards, sodium tetrachloropalladate (II) (Na_2_PdCl_4_) were slowly dropped into the solution. By controlling the dropping rate of Na_2_PdCl_4_, the forming rate of the Pd clusters is slow enough to avoid the formation of Pd NPs with large diameter and the slow formation of Pd clusters may assist the deposition of Pd on the Au seeds. Then *Cacumen Platycladi* (*C. Platycladi*) leaf extract were added, which acted as both weak reductant and capping agent. Capping agent is a key factor which not only imparts stability to the products but also controls morphology of the evolved particles [23]. After reaction of 30 min at room temperature, the Au-Pd core-shell NPs were obtained. In this experiment, low temperature is beneficial to avoid thermally diffusion of the atoms between the core and the outer shell. In addition, at lower temperature in *C. Platycladi* extract, the nucleation rate of Pd is slow, facilitating the formation of a fine flower-shaped core-shell structure. Interestingly, in our previous research, flower-shaped Au-Pd alloy NPs were obtained if HAuCl_4_ and Na_2_PdCl_4_ were reduced simultaneously with AA and *C. Platycladi* extract at room temperature. Moreover, alloy NPs were harvested only when AA was injected into the reaction solution prior to the *C. Platycladi* extract (Figure 1A) [24]. Hence, the structure of the NPs is highly dependent on the synthesis procedure.

Figure 1A presents the representative TEM images of the as-synthesized Au-Pd bimetallic NPs with initial Au/Pd ratio of 1:1, and the average size of as-prepared NPs was 47.8 ± 2.3 nm (the inset in Figure 1A). From the high-resolution TEM images (Figure 1B,C), a dark core surrounded by a light-color shell can be clearly distinguished, and the edges of the particles were not smooth instead of a flower-like structure at the periphery, indicating a core-shell structure of the nanoflowers (NFs) as expected. The *d*-spacing of the adjacent (111) lattice of the inner core part is 0.229 nm and the flower layer part is 0.225 nm (shown in Figure 1D), corresponding to the mean value of the (111) planes of face-centered cubic (*fcc*) Au and Pd, respectively [25].

### 2.2. SAED and XRD Analysis

**Figure 2.**
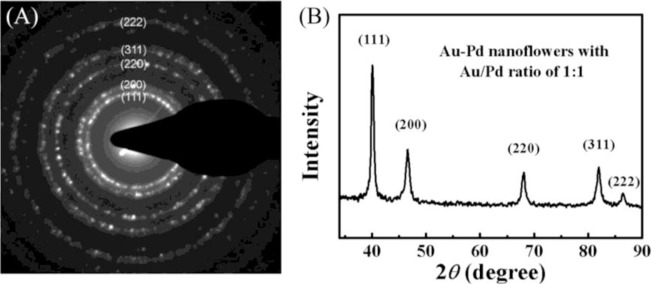
(**A**) SAED and (**B**) XRD patterns of as-prepared NPs with initial Au/Pd molar ratio of 1:1.

The SAED patterns were collected from a single NF (Figure 2A). We obtained diffraction patterns of individual particle by concentrating the beam while still keeping it parallel, which enabled us to identify the crystalline system, precisely measure the lattice parameters. The observed diffraction rings can be assigned to the (111), (200), (220), (311) and (222) diffractions of metal with face-centered cubic (*fcc*) structure. XRD patterns of the bimetallic NFs are presented in Figure 2B and the observed five peaks corresponding to five different crystal planes on the flower-shaped structure, consistent with the SAED data, suggesting that the NPs have features of polycrystalline.

### 2.3. UV-Vis Analysis

**Figure 3.**
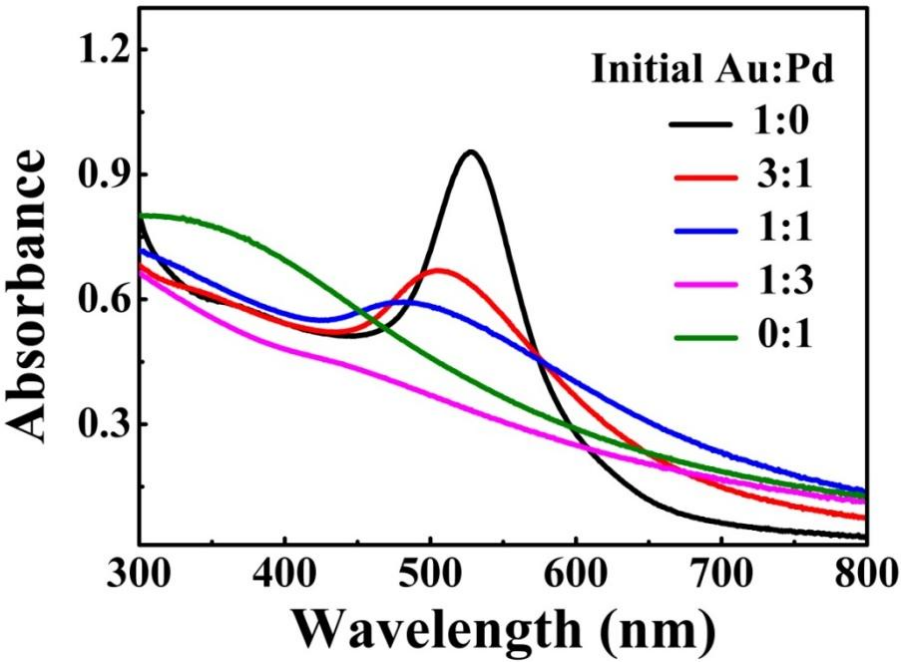
UV-Vis spectra of the as-prepared Au-Pd core-shell NFs with different initial Au/Pd molar ratios in water, which shows the single SPR peak assigned to the dipole resonance of Au core.

The metal NPs with specific geometrical shapes exhibit distinct plasmon absorption that is dependent on their sizes and shapes [26]. Figure 3 shows the extinction spectra of the as-prepared Au-Pd NFs in water, which shows the single surface plasmon resonance (SPR) peak that can be assigned to the dipole resonance of Au core. In addition, as the relative content of Pd increased, the SPR peak of Au-core quickly fades away. As previously reported [27], shells of Pd or Pt strongly damp out the dipolar plasmon oscillations of Au or Ag cores, because Pd or Pt have significantly lower conductivities at optical frequency than those of Au or Ag. As such, increase in shell thickness progressively damps out the SPR peak of the core. In our case, similarly, the Au core gave a single SPR peak centered at 530 nm but no peak was observed for the Pd petals [28]. For Au-Pd bimetallic NFs, with increasing the concentration of Pd the intensity of SPR peak of Au-core fades away.

### 2.4. EDX Elemental Analysis

**Figure 4.**
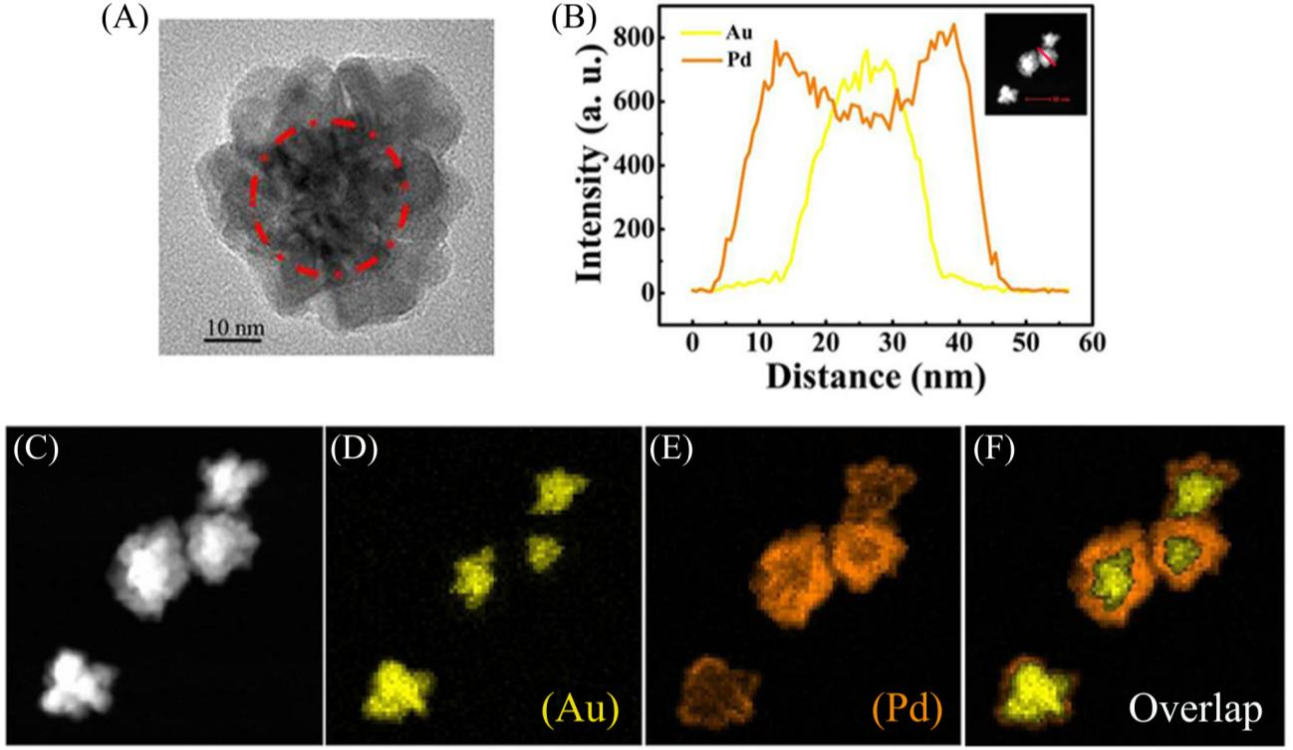
(**A**) HRTEM image of a single Au@Pd NF; (**B**) distribution of Au and Pd components along the cross-sectional line profiles of a single Au@Pd NF; (**C**) High-magnification STEM image of Au@Pd NF with Au/Pd molar ratio of 1:1; EDX elemental maps of (**D**) Au, (**E**) Pd concentrations in the NF and (**F**) overlap image of Au and Pd mapping.

To confirm the structure of the prepared NFs, the scanning transmission electron microscopy (STEM)-EDX images of the Au-Pd NFs are given in Figure 4.

Figure 4A shows a HRTEM image of an Au-Pd NF consisting of an Au core and Pd petals and the Pd petals consist of different crystalline domains. The line profiles of the composition on a single particle (Figure 4B) and the high-magnification STEM image (Figure 4C) show that the core-shell nanostructure consists of the Au NP as a core and a complete shell of Pd. Furthermore, elemental mapping of Au and Pd (Figure 4D–F) also reveal the core-shell structure of Au-Pd NFs. The colored elemental mapping images indicate that the Au atoms (yellow, Figure 4D) are distributed in the interior of the NFs and that of the Pd atoms (orange, Figure 4E) are deposited on the surface of Au core.

### 2.5. SERS of Rhodamine 6G

**Figure 5.**
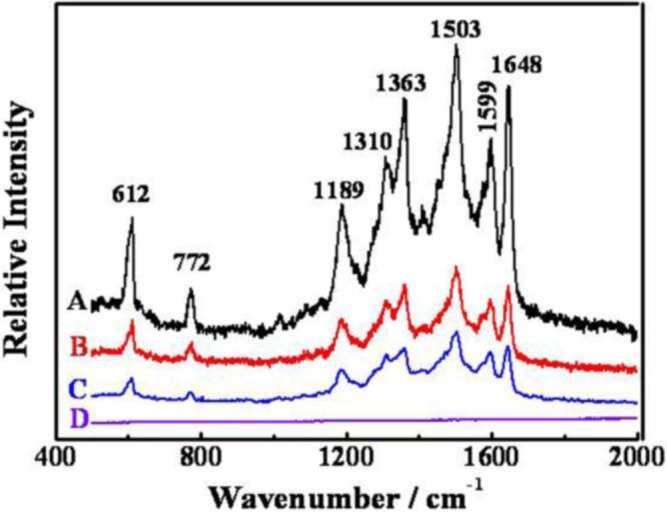
Raman spectra of (**A**) as-synthesized Au-Pd core-shell NFs with the initial Au/Pd molar ratio of 1:1; (**B**) Au NPs; (**C**) Pd NPs and (**D**) glass substrate with none adsorbed with 1 × 10^−8^ mol·L^−1^ R 6G.

The Au-Pd core-shell NFs with petalages surfaces might be good substrates for studying the SERS activity. Due to the large scattering cross section, R6G is chosen as the molecule probe in this SERS experiment [29]. As shown in Figure 5, the SERS spectrum obtained from the Au-Pd core-shell NFs (Figure 5A) absorbed with R6G solution (1 × 10^−8^ mol·L^−1^) is well presented. In comparison with the Raman scattering spectra of R6G solution (1 × 10^−8^ mol·L^−1^) without Au-Pd core-shell NFs (Figure 5D) and it is clear that R6G on the glass substrate of the Au-Pd core-shell NFs had strong enhancements at 612 cm^−1^, 772 cm^−1^, 1189 cm^−1^, 1310 cm^−1^, 1363 cm^−1^, 1503 cm^−1^, 1599 cm^−1^ and 1648 cm^−1^. We also replaced Au-Pd core-shell NFs by Au [30] and Pd [28] NPs which were both synthesized by *C. Platycladi* extract as the SERS substrate to compare their SERS properties, respectively (Figure 5B,C). It is obvious that R6G on the Au-Pd core-shell NFs has a stronger enhancement than that of R6G on the Au and Pd NPs. It might be due to the hierarchical and polycrystalline structures of the flower-like Au-Pd core-shell NPs, which gave some defects in their crystal structure, and the synergistic effect between Au-core and Pd-shell.

## 3. Experimental Section

### 3.1. Materials and Reagents

To obtain the *C. Platycladi* leaf extract (1g/100mL), 1 g of the screened *C. Platycladi* leaf powder (Xiamen Jiuding Drugstore, Xiamen, China) was dispersed in 100 mL deionized (DI) water, then placed in a water bath shaker at 30 °C for 4 h. The mixture was filtered to remove the residual insoluble biomass, and the resulting filtrate was used for the subsequent NPs synthesis. Ascorbic acid (AA), chloroauric acid (HAuCl_4_), sodium tetrachloropalladate (II) (Na_2_PdCl_4_), rhodamine 6G (R6G) and other chemical reagents were purchased from Sinopharm Chemical Reagent Co. Ltd (Guangzhou, China) and used as received. All glassware was cleaned with aqua regia and rinsed several times with DI water.

### 3.2. Synthesis of Flower-Like Au-Pd Core-Shell Nanoparticles

In a typical synthesis of flower-like Au-Pd core-shell NPs, 1 mL of 5 mM HAuCl_4_ was added to 40 mL DI water at room temperature, then 1 mL of 0.1 M AA and 5 min later, 1 mL of 5 mM Na_2_PdCl_4_ were slowly dropped into the solution in 10 min while stirring and 20 s later, 10 mL *C. Platycladi* extract (1 g/100 mL) were successively added to the mixture under vigorous stirring for another 15 min. For comparison, Au and Pd monometallic NPs were prepared in the same way by substituting aqueous solutions of HAuCl_4_ and Na_2_PdCl_4_ mixtures by HAuCl_4_ and Na_2_PdCl_4_, respectively.

### 3.3. Characterization of Au@Pd Core-Shell Nanoparticles

TEM observations, SAED analysis, and EDX analysis were performed on an electron microscope (Tecnai F30, FEI, Eindhoven, The Netherlands) with an accelerating voltage of 300 kV, where the samples were prepared by dipping the copper grid in the NPs hydrosol and allowing the water to evaporate. XRD measurements for the dried and powdered samples were conducted on an X-ray diffractometer (PANalytical BV, Phillips, Eindhoven, The Netherlands) equipped with Cu Kα radiation (40 kV, 30 mA). UV-Vis spectra were recorded on a spectrophotometer (Evolution-220, Pgeneral, Shanghai, China) using a 1 cm quartz cell from hydrosol samples.

### 3.4. Raman Detection

After the flower-shaped Au-Pd core-shell NPs were washed with DI water, 0.005 g of the composites were dried on a glass slide, and 30 μL (10^−8^ M) of R6G was dropped on the flower-shaped Au-Pd core-shell NPs for Raman measurement. The Raman spectra were recorded using a Renishaw in Via Raman system with a 632.8 nm He-Ne laser as the excitation source. The laser power that reached the sample was 0.4 mW. The spectra acquisition time was 10 s. The spectra were based on an average result of three measurements.

## 4. Conclusions

In conclusion, we have developed a method to synthesize the Au@Pd NFs involving the successive reduction of AuCl_4_^−^ and PdCl_4_^2−^ assisted by *Cacumen Platycladi* leaf extract. During the synthesis, low temperature and slow forming rate of the Pd clusters are vital to facilitate the formation of fine flower-like core-shell nanoparticles. Energy-dispersive X-ray spectroscopy, UV-Vis spectroscopy, and X-ray diffraction characterizations confirmed the nature of the core-shell structure. The obtained flower-shaped Au-Pd core-shell NPs also performed excellent SERS enhancement.
